# Lymphogranulomatous prostatitis due to coccidioidomycosis: A rare complication of disseminated disease

**DOI:** 10.1016/j.mmcr.2026.100832

**Published:** 2026-10-27

**Authors:** Ricardo Olivas Lerma, Rodolfo Myronn de Melo Rodrigues, Reyes Castanon, Jyothsna Goranti, Jeffrey Sherwood

**Affiliations:** Texas Tech University Health Sciences Center at El Paso, Paul L. Foster School of Medicine, United States

**Keywords:** Coccidioidomycosis, Prostatitis, Genitourinary infection, Disseminated disease, Blastomycosis

## Abstract

Prostatic coccidioidomycosis is an exceptionally rare manifestation of disseminated infection. A 48-year-old man with diabetes and prior pulmonary coccidioidomycosis developed recurrent cavitary pneumonia and urinary retention. Prostate biopsy demonstrated lymphogranulomatous inflammation and fungal spherules. Serial complement-fixation titers ranged from 1:64 to 1:128. *Coccidioides* was not isolated in culture; therefore, species-level identification was not possible. Approximately 9 months after prostate biopsy, the patient was voiding independently while receiving chronic fluconazole 400 mg daily. This case emphasizes prolonged suppressive therapy, serologic monitoring, and distinction between coccidioidal prostatitis and blastomycosis.

## 1 Introduction

Coccidioidomycosis was first described in 1892 in an Argentine patient with progressive cutaneous disease [1]. The causative dimorphic fungi, *Coccidioides immitis* and *Coccidioides posadasii*, produce clinically indistinguishable infections but have partially distinct geographic distributions. *C. immitis* predominates in California and parts of the western United States, whereas *C. posadasii* occurs predominantly in Arizona, Texas, other areas of the southwestern United States, Mexico, and Central and South America; geographic overlap prevents reliable species identification from location alone [2].

In the environmental phase, *Coccidioides* grows as hyphae that produce infectious arthroconidia. Disturbance of endemic soil, particularly near small-mammal burrows, can aerosolize arthroconidia and permit inhalational exposure [3], [4]. The primary infection is pulmonary: inhaled arthroconidia enter the lungs and develop into spherules containing endospores. Rupture of mature spherules releases endospores that produce additional spherules within host tissues, and hematogenous spread from the pulmonary focus can establish extrapulmonary disease [2], [5]. Mature spherules containing endospores are a characteristic, pathognomonic histopathologic finding; imaging findings are nonspecific and are not pathognomonic [2].

Approximately 60% of individuals exposed to *Coccidioides* remain asymptomatic. Symptomatic pulmonary infection commonly resembles community-acquired pneumonia, with fever, cough, chest pain, or dyspnea, and radiography may demonstrate infiltrates, adenopathy, nodules, or cavitation [2], [5]. Extrapulmonary dissemination occurs in approximately 0.5–2.0% of infected persons, most often involving the skin, bones, joints, or meninges [2]. Genitourinary involvement is distinctly uncommon; in men, reported sites include the prostate, epididymis, seminal vesicles, and regional lymph nodes [6], [7], [8]. Coccidioidal prostatitis may cause urinary retention, prostatic enlargement, lower urinary tract symptoms, or increased prostate-specific antigen, and has often been diagnosed after biopsy or resection performed for suspected benign prostatic hyperplasia or malignancy [6], [7], [8], [9], [10], [11].

When antifungal treatment is indicated, oral fluconazole is the usual first-line agent for most clinically significant pulmonary and nonmeningeal extrapulmonary disease; amphotericin B is reserved for severe, rapidly progressive, refractory, or otherwise complicated infection [12].

We report a patient with diabetes mellitus and recurrent cavitary pulmonary coccidioidomycosis whose persistent urinary symptoms led to the diagnosis of lymphogranulomatous prostatitis due to disseminated *Coccidioides* infection.

## 2 Case presentation:

A 48-year-old man residing in El Paso, Texas, had diabetes mellitus complicated by peripheral neuropathy, a history of prior alcohol use disorder, and biopsy-supported pulmonary coccidioidomycosis diagnosed in 2016. At the initial diagnosis, lung tissue demonstrated granulomatous inflammation with fungal spherules consistent with *Coccidioides* infection, and he completed a 1-year course of fluconazole. The available infectious-disease documentation did not specify the diabetes type. A contemporaneous glycated hemoglobin (HbA1c) result was not available in the records provided; consequently, the degree of glycemic control at recurrence could not be established.

In December 2024, he was hospitalized with acute hemoptysis, fever, and dyspnea. Day 0 was defined as December 16, 2024, the date of the first documented computed tomography during evaluation of the recurrent pulmonary illness. Chest imaging demonstrated bilateral patchy airspace and cavitary abnormalities, with chronic cavitary disease most prominent in the left upper lobe (Fig. 1). *Coccidioides* immunoglobulin M and immunoglobulin G serology was positive on December 17 and December 19, 2024. Bronchoalveolar lavage performed on day 3 (December 19, 2024) yielded normal respiratory flora on bacterial culture; fungal culture grew *Candida glabrata* but did not isolate *Coccidioides*. Specifically, Grocott methenamine silver staining was performed on the bronchoalveolar lavage cytology specimen and highlighted fungal elements consistent with *Coccidioides*. Acid-fast bacillus studies were negative. Oral fluconazole 400 mg once daily was restarted, with subsequent improvement in respiratory symptoms.

**Fig. 1:**
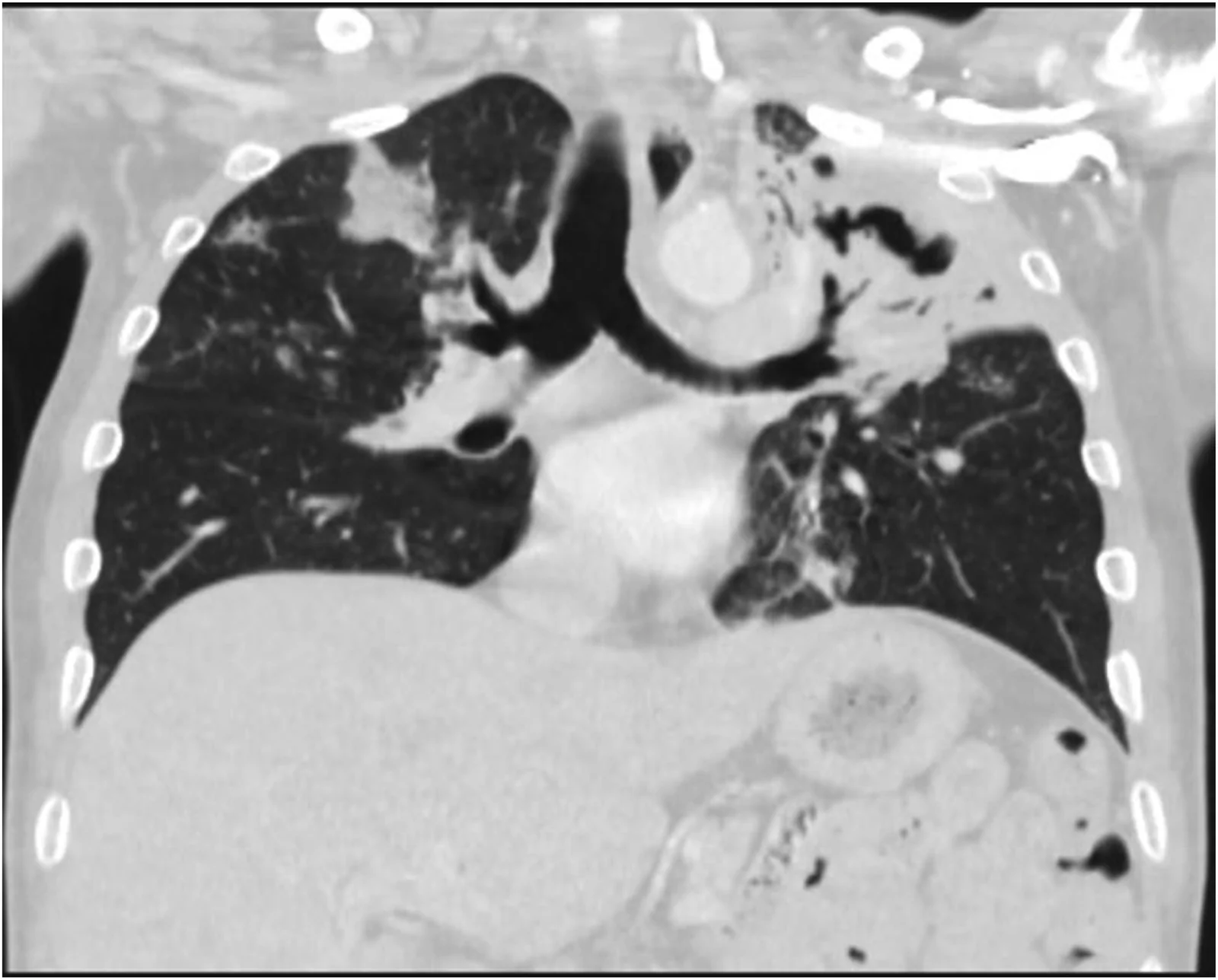
CT chest demonstrating chronic cavitary pulmonary disease most notable in the left upper lobe.

During the same hospitalization, he also reported lower abdominal discomfort, difficulty voiding, and urinary retention. Urinalysis showed no significant pyuria, and the urine culture was negative for bacterial growth. Abdominal and pelvic computed tomography demonstrated a markedly distended urinary bladder and an enlarged prostate containing areas of decreased attenuation (Fig. 2). These imaging findings indicated prostatic pathology but were not specific for a fungal infection.

**Fig. 2:**
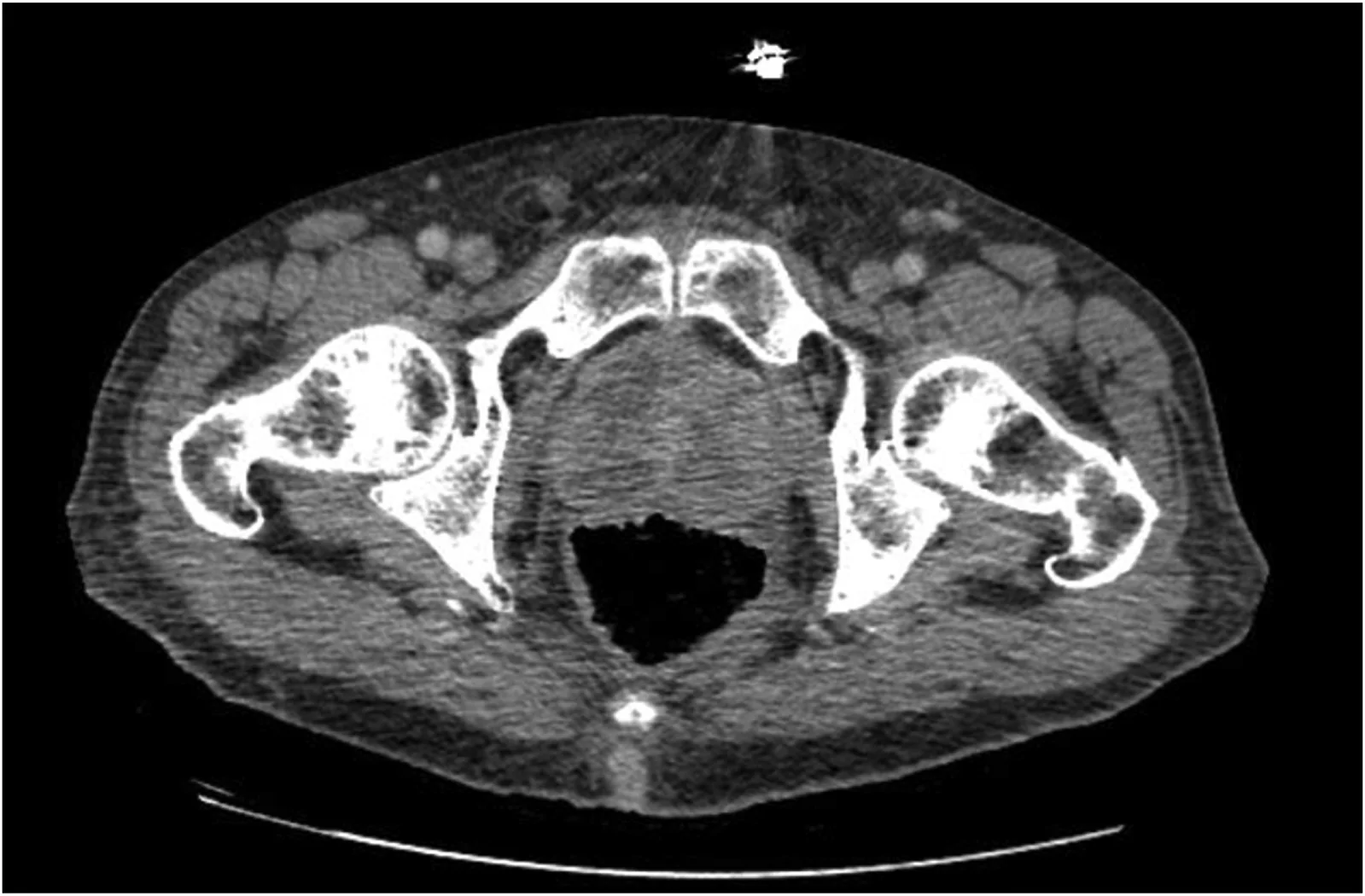
CT abdomen and pelvis showing enlarged prostate with areas of decreased attenuation and urinary retention.

On day 144 (May 9, 2025), *Coccidioides* immunodiffusion was positive, and the complement-fixation titer was elevated at 1:64. Follow-up chest computed tomography in June 2025 showed persistent left upper-lobe scarring, cavitation, and traction bronchiectasis. Despite improvement in pulmonary symptoms while receiving fluconazole, lower urinary tract dysfunction persisted and initially required tamsulosin and intermittent self-catheterization, prompting referral to urology.

In September 2025, transrectal ultrasound-guided prostate biopsy demonstrated lymphogranulomatous prostatitis with fungal elements morphologically consistent with coccidioidal spherules in multiple hematoxylin-and-eosin-stained cores (Fig. 3). Periodic acid–Schiff staining of the prostatic tissue further highlighted these fungal elements (Fig. 4). No malignancy was identified. In contrast to the Grocott methenamine silver stain used on the respiratory cytology specimen, hematoxylin and eosin and periodic acid–Schiff stains were used to evaluate the prostatic biopsy. The exact biopsy day within September was not documented.

**Fig. 3:**
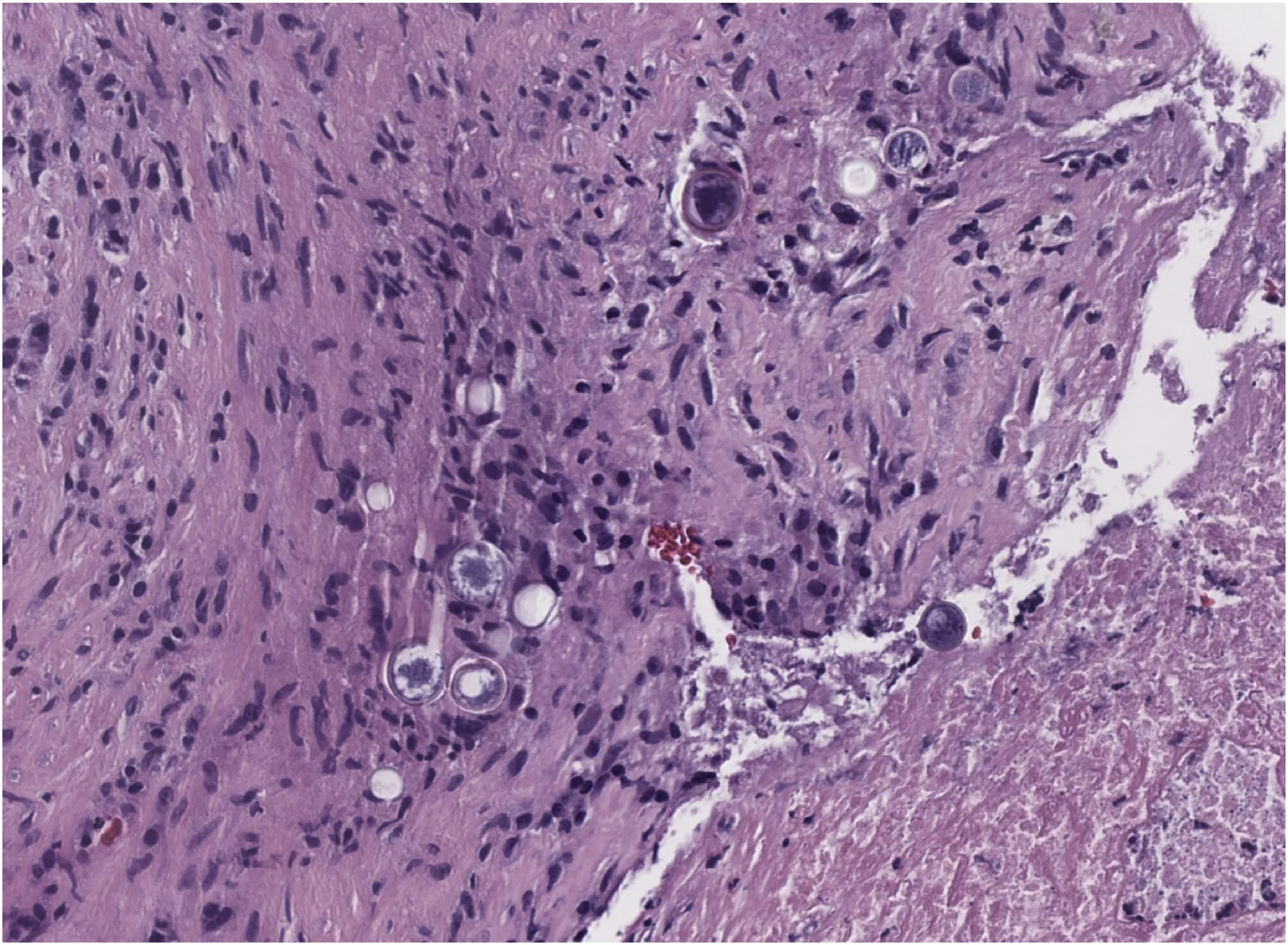
Prostate biopsy, H&E stain showing lymphogranulomatous inflammation with fungal spherules consistent with *Coccidioides* spp.

**Fig. 4:**
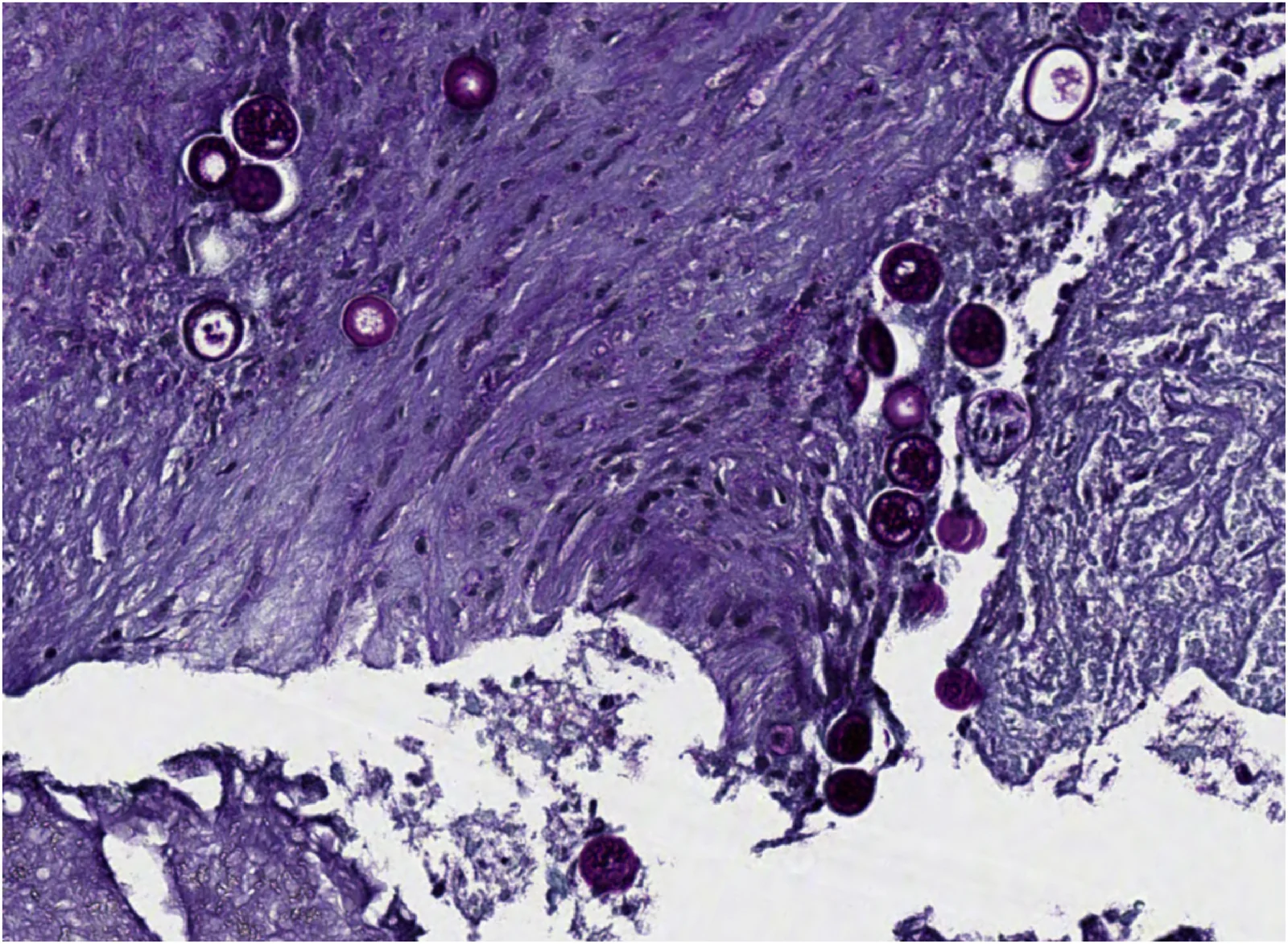
PAS fungal stain also highlighting fungal elements consistent with *Coccidioides* spp.

Serial complement-fixation titers remained elevated: 1:64 on May 9, 2025; 1:128 on day 361 (December 12, 2025); and 1:64 on day 478 (April 8, 2026), with positive immunodiffusion results. No *Coccidioides* isolate was recovered, and molecular species identification was not documented; therefore, the infection could not be assigned to *C. immitis* or *C. posadasii*. Although the clinical laboratory referred to the assay as a “*C. immitis* complement-fixation” test, this serologic label does not establish the infecting species. In April 2026, recurrent hemoptysis prompted repeat chest imaging, which showed pulmonary nodules, bronchiectasis, and ground-glass opacities without pulmonary embolism; the patient received a 1-week course of amoxicillin-clavulanate and doxycycline for possible superimposed bacterial pneumonia.

At the most recent documented infectious-disease follow-up on day 543 (June 12, 2026), approximately 9 months after the September 2025 prostate biopsy and approximately 18 months after the December 2024 recurrence, the patient remained on fluconazole 400 mg orally once daily. He reported no recurrent hemoptysis, improving respiratory symptoms, and independent urination while taking tamsulosin; a recent symptomatic urinary tract infection had been treated with trimethoprim-sulfamethoxazole. Given prior relapse, extrapulmonary dissemination, and persistently elevated titers, the infectious-disease specialist recommended continued chronic suppressive fluconazole with repeat clinical assessment in 3 months. The clinician documented that suppression might continue until complement-fixation titers are consistently below 1:4, while recognizing that the duration may be prolonged or lifelong. Ongoing follow-up should include serial titers, surveillance of pulmonary and genitourinary symptoms, medication-safety monitoring, and assessment and optimization of glycemic control.

## 3 Discussion

Prostatic coccidioidomycosis is an exceptionally uncommon manifestation of disseminated infection, potentially reflecting under-recognition as well as true rarity. In a review of 3676 prostate pathology reports from Arizona, 44 specimens showed granulomatous prostatitis, but only 4 demonstrated coccidioidal infection, corresponding to approximately 0.1% of all reviewed prostate specimens [8]. Although the prostate appears repeatedly among reported male genitourinary sites, its involvement remains exceptional in the overall clinical spectrum of coccidioidomycosis [6], [7], [8], [9], [10], [11].

Dissemination always follows an initial pulmonary infection and occurs through hematogenous spread from the lungs. Risk is increased by impaired cell-mediated immunity, diabetes mellitus, male sex, pregnancy, African or Filipino ancestry, and immunosuppressive conditions or therapies [2], [12], [13]. In the present case, long-standing diabetes and recurrent cavitary pulmonary disease supported concern for complicated infection. However, because the diabetes type and a contemporaneous HbA1c measurement were not documented, neither diabetes subtype nor the level of glycemic control can be characterized; the missing HbA1c limits assessment of the potential contribution of hyperglycemia.

Genitourinary coccidioidomycosis can mimic benign prostatic hyperplasia, bacterial prostatitis, prostatic abscess, or malignancy. Urinary retention, obstructive lower urinary tract symptoms, prostate enlargement, and elevated prostate-specific antigen are clinically nonspecific, and cross-sectional imaging cannot distinguish coccidioidal prostatitis from other inflammatory or neoplastic conditions [6], [7], [8], [9], [10], [11]. Persistent urinary symptoms in a patient with pulmonary coccidioidomycosis should therefore prompt urologic evaluation and consideration of tissue sampling.

Histopathologic identification of coccidioidal spherules in granulomatous or lymphogranulomatous inflammation provides the key diagnostic evidence. Hematoxylin and eosin, periodic acid–Schiff, and Grocott methenamine silver stains can facilitate recognition of fungal elements in the appropriate specimen [2], [6], [8]. Elevated *Coccidioides* complement-fixation titers support the assessment of disease burden and complicated infection but do not independently establish dissemination. In a study of 434 antifungal-treated patients, median maximal titers were 1:4 in uncomplicated pulmonary disease, 1:24 in chronic pulmonary disease, and 1:128 in nonmeningeal disseminated disease; a threshold of at least 1:32 showed moderate discriminatory value for complicated infection [14]. In this patient, serial titers increased from 1:64 in May 2025 to 1:128 in December 2025 and subsequently decreased to 1:64 in April 2026, documenting persistent serologic activity despite a later decline. The prostate biopsy, rather than the titer alone, established the extrapulmonary site. Serial titers should be interpreted alongside symptoms, imaging, and longitudinal clinical findings.

A limitation of this case is the absence of a recovered *Coccidioides* isolate and documented molecular identification. Bronchoalveolar lavage fungal culture grew *Candida glabrata*, but *Coccidioides* was identified only by compatible cytomorphology and serology. Consequently, the infecting species cannot be determined as either *C. immitis* or *C. posadasii*. A laboratory label referring to a “*C. immitis* complement-fixation” assay denotes serologic testing and does not constitute species-level identification of the clinical infection. Although *C. posadasii* may be encountered in West Texas, geographic residence alone is also insufficient for definitive species assignment. The absence of species-level identification does not negate the diagnosis because the characteristic tissue morphology, compatible serologic profile, and established pulmonary infection converge on coccidioidomycosis.

Blastomycosis is an essential alternative endemic fungal diagnosis when pulmonary disease is accompanied by prostatitis in the United States. Unlike coccidioidomycosis, for which prostatic disease is exceptional, disseminated blastomycosis has a recognized predilection for the male genitourinary tract. Genitourinary involvement has been described in approximately 10–30% of blastomycosis cases, and the prostate and epididymis are among the most frequently involved sites [15]. Prostatic blastomycosis can manifest with urinary obstruction, prostatitis, or abscess formation, including cases confirmed through isolation of *Blastomyces* from genitourinary specimens [16]. Exposure geography also differs: *Coccidioides* is associated predominantly with the arid southwestern United States, whereas *Blastomyces* is classically associated with the Ohio and Mississippi River valleys, Great Lakes region, and other moist environments [2], [15].

Tissue morphology distinguishes these infections: *Coccidioides* produces nonbudding spherules that may contain endospores, whereas *Blastomyces* appears as thick-walled yeast with broad-based budding [15]. Therefore, residence in West Texas, recurrent pulmonary coccidioidomycosis, positive *Coccidioides* serology, and spherules in the prostatic biopsy favored coccidioidal rather than blastomycotic prostatitis in this patient. Clinical manifestations and imaging alone cannot reliably separate the two infections.

Management of coccidioidal prostatitis is informed by case reports, expert consensus, and guidelines for nonmeningeal extrapulmonary disease rather than site-specific randomized trials [8], [12], [17]. Fluconazole is the preferred initial oral agent for most stable patients, whereas amphotericin B is reserved for severe, refractory, or rapidly progressive infection [12], [17]. By June 2026, this patient had received approximately 18 months of fluconazole following the December 2024 recurrence. A previous relapse after a completed year of fluconazole, documented prostatic dissemination, diabetes, and titers that remained at or above 1:64 support continued long-term and potentially lifelong azole suppression. The treating infectious-disease specialist planned serial 3-month clinical assessments and continued therapy potentially until complement-fixation titers are consistently below 1:4. This titer target reflects an individualized clinical monitoring plan rather than a validated universal threshold for safely stopping therapy. Sustained follow-up over many years should include serial titers, assessment of urinary and pulmonary symptoms, monitoring for fluconazole toxicity and drug interactions, and optimization of diabetes management [12], [14], [17].

These findings highlight the need to investigate persistent urinary symptoms in patients with pulmonary coccidioidomycosis, particularly when diabetes or recurrent cavitary disease increases concern for complicated infection. Coordinated assessment by infectious diseases, urology, radiology, and pathology can identify an otherwise overlooked extrapulmonary focus and guide prolonged treatment.

## 4 Conclusion

Lymphogranulomatous prostatitis due to disseminated coccidioidomycosis is an exceptionally rare but clinically important cause of urinary obstruction in endemic regions. In this patient, recurrent pulmonary infection, an elevated complement-fixation titer, and characteristic prostatic fungal spherules established the diagnosis despite the absence of a *Coccidioides* isolate or species-level identification. Distinguishing coccidioidal prostatitis from the more familiar genitourinary manifestations of blastomycosis requires attention to exposure history, serology, and tissue morphology. Documented improvement approximately 9 months after prostate biopsy supports continued prolonged, potentially lifelong fluconazole suppression with long-term clinical and serologic surveillance.

## CRediT authorship contribution statement

**Ricardo Olivas Lerma:** Writing – review & editing, Writing – original draft, Investigation, Data curation, Conceptualization. **Rodolfo Myronn de Melo Rodrigues:** Writing – review & editing, Writing – original draft. **Reyes Castanon:** Writing – original draft, Investigation. **Jyothsna Goranti:** Writing – review & editing, Writing – original draft. **Jeffrey Sherwood:** Writing – review & editing, Supervision.

## Ethical form

*Medical Mycology Case Reports* requires full disclosure of all sources of funding and potential conflicts of interest. The journal also requires a declaration that the author(s) have obtained written and signed consent to publish the case report from the patient or legal guardian(s).

If you have nothing to declare in any of these categories then this should be stated.

## Consent

Please declare that you have obtained written and signed consent to publish the case report from the patient or legal guardian(s).

Please state that consent has been obtained from the patient or legal guardian(s)

Written informed consent was obtained from the patient or legal guardian(s) for publication of this case report and accompanying images. A copy of the written consent is available for review by the Editor-in-Chief of this journal on request.

As corresponding author, I hereby declare that I sign this document on behalf of all the authors of the above mentioned manuscript.

## Funding source

All sources of funding should be acknowledged and you should declare any extra funding you have received for academic research of this work. If there are none state ‘there are none’.

**Please state any sources of** funding **for your research**

There are none.

## Conflict of interest

No financial support or conflicts of interest exist.
